# Hyaluronan-Phosphatidylethanolamine Polymers Form Pericellular Coats on Keratinocytes and Promote Basal Keratinocyte Proliferation

**DOI:** 10.1155/2014/727459

**Published:** 2014-09-09

**Authors:** Caitlin J. Symonette, Aman Kaur Mann, Xiao Cherie Tan, Cornelia Tolg, Jenny Ma, Francisco Perera, Arjang Yazdani, Eva A. Turley

**Affiliations:** ^1^Division of Plastic and Reconstructive Surgery, University of Western Ontario, 1151 Richmond Street, London, ON, Canada N6A 3K7; ^2^London Regional Cancer Program, London Health Sciences Centre, Room A4-931A, 790 Commissioners Road East, London, ON, Canada N6A 4L6; ^3^London Health Science Center, Victoria Hospital, Room E2-647, 800 Commissioners Road East, London, ON, Canada N6A 4G5

## Abstract

Aged keratinocytes have diminished proliferative capacity and hyaluronan (HA) cell coats, which are losses that contribute to atrophic skin characterized by reduced barrier and repair functions. We formulated HA-phospholipid (phosphatidylethanolamine, HA-PE) polymers that form pericellular coats around cultured dermal fibroblasts independently of CD44 or RHAMM display. We investigated the ability of these HA-PE polymers to penetrate into aged mouse skin and restore epidermal function in vivo. Topically applied Alexa^647^-HA-PE penetrated into the epidermis and dermis, where it associated with both keratinocytes and fibroblasts. In contrast, Alexa^647^-HA was largely retained in the outer cornified layer of the epidermis and quantification of fluorescence confirmed that significantly more Alexa^647^-HA-PE penetrated into and was retained within the epidermis than Alexa^647^-HA. Multiple topical applications of HA-PE to shaved mouse skin significantly stimulated basal keratinocyte proliferation and epidermal thickness compared to HA or vehicle cream alone. HA-PE had no detectable effect on keratinocyte differentiation and did not promote local or systemic inflammation. These effects of HA-PE polymers are similar to those reported for endogenous epidermal HA in youthful skin and show that topical application of HA-PE polymers can restore some of the impaired functions of aged epidermis.

## 1. Introduction

Hyaluronan (HA) is a ubiquitous extracellular matrix tissue polysaccharide belonging to the glycosaminoglycan family, which is characterized by repeating hexosamines and uronic acid [1–3]. Skin HA accounts for approximately 50% of total body HA and occurs in both the epidermal, and dermal layers. Here it performs a variety of functions that are related to its rheological, viscoelastic and biological properties [4–6]. For example, its rheological properties contribute to the overall quality, hydration, permeability, and immune barrier functions of skin while its unique viscoelastic properties protect skin cells from mechanical damage [7–11]. The biological properties of HA include a contribution to cell survival, proliferation, and migration, and result from its ability to activate key signaling cascades through interactions with cellular HA receptors, which in keratinocytes is primarily CD44 [12–18]. HA is also an important regulator of skin immune surveillance [19] and response to injury processes [15, 20–25]. Although the primary structure of HA is simple and is composed only of linear repeating N-acetyl-D-glucosamine and glucuronic acid disaccharides, its functions are complexly regulated and dependent upon its organization by extracellular and cellular proteins as well as by polymer size [14, 16, 21, 23].

The organization of HA in the extracellular matrix and around cells is a critical component of its skin functions [6, 26, 27]. On keratinocytes, HA is structured as compact pericellular coats that are maintained by CD44. Dermal HA is more abundantly extracellular and linked to a variety of proteoglycans including versican [28–34]. The cellular and extracellular organization of skin HA is critical for its retention in the papillary dermal and keratinocyte layers. Quantitative loss of HA from these layers is associated with skin pathologies including poor wound healing, reduced skin elasticity/mobility, and loss of keratinocyte tight-junctions and permeability barrier functions [4, 27, 29, 35–44]. Polymer size also contributes to the skin functions of HA. For example, high molecular weight (HMW), native HA in skin protects against tumor initiation [45], provides intrinsic water binding properties of skin [26] and is required for dendritic cell functions [15, 19, 46]. It regulates the proliferation and differentiation of the basal keratinocyte layer during homeostasis and response to injury [4, 6, 47, 48] and contributes to the barrier/hydration function [26, 34, 49] and structure of the stratum corneum [50].

Most HA in homeostatic skin is high molecular weight but fragmentation occurs following injury or prolonged exposure to UV. In cooperation with the fragmentation of other extracellular matrix components, HA fragments activate signaling cascades in keratinocytes and dermal fibroblasts that control migration, survival, and redifferentiation required for repair of injured skin [51–58]. HA fragments are also key regulators of innate immunity and are required for in-trafficking and proinflammatory cytokine expression of macrophages [15, 19]. The different functional effects of native versus fragmented HA likely result from selective interactions with specific receptors and differential effects of polymer size on the clustering/signal activation through these receptors [13, 15, 59]. The effects of native HA on homeostatic keratinocyte functions are mediated through CD44 [4, 30] while repair functions of HA fragments involve coordination of signaling through CD44 : RHAMM and TLR2, 4 complexes [55, 60, 61].

Chronological skin aging results in physiological alterations of keratinocytes and epidermal functions that contribute to epidermal thinning or atrophy, and barrier dysfunction, delayed wound repair, as well as increased susceptibility to pathologies including ulceration, dermatitis, and eczema [4, 60, 62]. Although age-associated epidermal dysfunction is not well understood, it is associated with changes in HA concentration and organization, and CD44 display [4, 63]. Experimental models have established that reduction or loss of keratinocyte CD44 results in epidermal changes that are similar to aging dysfunction such as thinning of the epidermal layer, barrier dysfunction, modified HA metabolism, reduced HA production, altered keratinocyte differentiation, and decreased skin elasticity. Application of HA to aged mouse skin partially restores permeability barrier homeostasis and epidermal thickness [41, 42]. Topical or injected HMW HA products have had variable effects in restoring a sustained physiological and hydrated microenvironment of youthful skin required for optimizing tissue repair and rejuvenation [4, 62, 64, 65]. Transepidermal or dermal HA delivery modalities, although promising, have similarly failed to reliably replenish sustained high levels of native HMW HA in the epidermis or dermis of aged skin [66–68]. This failure is likely because of poor penetration of topically applied HA formulations of MW greater than 50 KDa [39, 69–71], reduced HA capture in the epidermis as a result of declining CD44 levels [4, 42, 72] and aberrant organization as well as rapid clearance of the exogenous HA formulations [40, 60].

To address this problem, we developed a cell-based screening method for identifying HA-phospholipid (phosphatidylethanolamine, HA-PE) formulations that form pericellular HA coats on fibroblasts and keratinocytes in a CD44-independent manner [73]. We show that topical administration of HA-PE to the shaved skin of aged wild type or CD44−/− mice increased HA within the epidermal layer. This HA modification promoted basal keratinocyte and hair follicle proliferation as well as increased epidermal thickness but does not detectably alter the differentiation of keratinocytes. In addition, topical application of HA-PE in vivo did not result in either a local or a systemic inflammatory response.

## 2. Methods

### 2.1. Animals

Forty-five retired breeder ten-month old female C57BL/6 mice (Jackson Laboratory) were used for the multiple cream application studies. An additional twelve retired breeder twelve-month old female C57BL/6 mice were used for the Alexa^647^ mouse experiments.

Animals were individually caged in a temperature-controlled environment with a 12 h light/dark cycle and fed a standard mouse chow diet. All experiments were approved by and compliant with the standard operating protocols of the Animal Use Subcommittee at the University of Western Ontario, Canada (2009-051).

### 2.2. Preparation of HA-PE and HA

For preparation of the HA-PE cream (Patent identification: WO2011140630 A1) [74], 1.35 mL of unrefined liquid soy lecithin (Soy Lecithin GT non-GM IP, Imperial-Oel-Import, Germany) was mixed thoroughly at room temperature with 1.35 mL of 1% v/w Sodium Hyaluronate Solution (500 kDa, Medical Grade, Lifecore Biomedical, Chaska, MN, USA) and 252 *μ*L of isopropanolol. Subsequently, 3.78 mg of dry 1-ethyl-3-(3-dimethylaminopropyl) carbodiimide (EDC) was added and mixed thoroughly as a linking agent. After mixing for 10–15 minutes, 3 mL of vehicle cream was added and mixed for another 5 minutes. For preparation of unmodified HA cream, 1.35 mL of 1% v/w Sodium Hyaluronate Solution was mixed thoroughly with 3 mL of vehicle cream.

A water-based cream (Mango Face cream, Aquatech, Toronto) was used throughout the study as the vehicle for mixing with unmodified HA or HA-PE. Formulated creams were stored protected from light at 4°C.

### 2.3. HA Pericellular Coat Detection

HA pericellular coats were detected using particle exclusion [75]. Dermal wild type, CD44−/−, RHAMM−/−, and CD44 : RHAMM−/− embryonic fibroblasts were plated onto 35 mm tissue culture dishes in DMEM + 10% FBS for 24 hrs. Cells were then fixed in 2.5% glutaraldehyde in 0.1 M cacodylate buffer, 5 mM CaCl_2_, pH 7.2, for 30 min then washed gently in cacodylate buffer. 1 mL of either FITC-labeled 0.4 mm microspheres (Invitrogen) or formalized sheep erythrocytes (1 × 10^8^ erythrocytes/mL) was added to each 35 mm culture dish and swirled gently so that cells were evenly covered. Dishes were incubated for 15–30 min at 37°C to let beads or erythrocytes settle around cells. As a control, cells were incubated with 200 *μ*g/mL bovine testicular hyaluronidase (Sigma) for 1 h at 37°C prior to performing the particle exclusion assay. Cells were then photographed with a Nikon inverted microscope equipped with epifluorescence and Hoffmann optics.

### 2.4. Preparation of A^647^-HA

In the preparation of A^647^-HA, the solution was protected from ambient light. In a laminar flow hood, 0.0028 g of EDC (Sigma Aldrich, http://www.sigmaaldrich.com/) was dissolved in a 1 mL solution of 20 mM MES and 30% ethyl alcohol (pH 4.5) in a 15 mL tube. Subsequently, 200 *μ*L of pharmaceutical grade 1% v/w Sodium Hyaluronate Solution (500 kDa) (Lifecore Biomedical, Chaska, MN, USA) was added. After five minutes, 300 *μ*L of Alexa Fluor 647 Hydrazide, Tris (triethylammonium) salt (Life Technologies, http://www.lifetechnologies.com/) was added. The A^647^-HA solution was placed on a rocker at room temperature for 12 hours and then dialyzed (10,000 dalton cut off, Thermoscientific) at 4°C. The entire volume of 1X PBS buffer was exchanged at 1, 3, and 5 days. The retained A^647^-HA solution was retrieved from the dialysis apparatus and stored in a 15 mL tube in the 4°C fridge.

### 2.5. Application of A^647^-HA/HA-PE to Mice

Mice were anesthetized using Isofluorane gas for application of cream. While anesthetized, the upper dorsum of the back was shaved with an electric razor, leaving a strip of hair in midline to define the right and left sides. 0.18 g of A^647^-HA and A^647^-HA-PE cream was applied to the left and right sides, respectively. Two mice were euthanized using a CO_2_ chamber at each of six time points: 40 min, 2 h, 4 h, 8 h, 24 h, and 72 h following cream application.

### 2.6. Preparation and Analysis of A^647^-HA-PE

A full-thickness biopsy of the A^647^-HA/HA-PE treated area was obtained and lightly fixed for 10 minutes in 1.5% paraformaldehyde containing 0.5% cetylpyridinium chloride monohydrate [75] in PBS. Each specimen was subsequently fixed in 4% paraformaldehyde (pH 7.4) for an additional 30 min then stored at −4°C. Tissue samples were paraffin processed, mounted then analyzed with a Nikon Eclipse motorized upright microscope. The penetration of A^647^-HA/HA-PE into the epidermis was quantified using Image J, which converted fluorescent images to pixel density per a tissue area. A total of twelve C57BL/6 retired breeder female mice were used for this experiment.

### 2.7. Treatment of Mice with HA-PE

Three treatment arms were used for these experiments: HA-PE cream, HA cream, and vehicle cream and 15 mice were used per group. For the initial cream application, mice were anesthetized, shaved, and treated as described above. Subsequent once daily cream applications were performed without anesthesia. Five mice in each treatment arm were sacrificed after one, five, and ten applications. At each of these times, mice were euthanized, a blood sample via a cardiac puncture and two four-millimeter punch skin biopsies was obtained. The remainder of the shaven treatment area was excised and stored at −80°C. To provide a positive control for inflammation markers, an additional mouse was wounded with a 4 mm punch biopsy as described previously [76], then tissue harvested using an 8 mm punch biopsy 3 days after wounding. All tissue biopsies were fixed in 4% paraformaldehyde/PBS at 4°C for 24 h. Samples were then processed for paraffin histology sections. For immunohistochemistry, tissue sections were deparaffinized and antigen retrieval was performed by heating tissue sections in a microwave oven in 0.01 M aqueous sodium citrate buffer (pH 6.0). Tissue sections were washed then incubated with one of the following primary antibodies: rabbit Ki67 monoclonal (1 : 100 Abcam), rabbit K10 monoclonal (1 : 7000 dilution, Abcam), and rat F4/80 monoclonal (1 : 100 dilution, AbD Serotec) followed by the appropriate biotinylated secondary antibody. Sections were counterstained with hematoxylin then mounted in Richard-Allan Scientific Cytoseal 60 (ThermoScientific). Digital images of stained tissue sections were obtained using an Aperio Scanscope. Five representative areas were taken per mouse and analyzed using Image J.

### 2.8. Measurement of Epidermal Thickness

To evaluate epidermal thickness, the above tissue sections stained with hematoxylin and eosin (4X magnification images) were analyzed using Image J. For each mouse, fifty serial measurements at 75 um intervals per section were made to determine epidermal thickness (*stratum basale* to the* stratum granulosum*) [77].

### 2.9. TNF-*α* ELISA

Activated macrophages and proinflammatory cytokines, such as TNF*α*, appear to be ubiquitous players in cutaneous inflammation regardless of the inciting stimulus whether wounding or sterile inflammation [78–80]. Dissected tissue samples from each of treatment and group and wounded tissue were mixed gently RIPA lysis buffer and a protease inhibitor tablet for 10 min and then sonicated for three 20-second pulses. Samples were incubated on a rotary shaker at 4°C in lysis buffer for an additional 45 min then centrifuged at 13,000 g (4°C) for 30 min. The pellet was discarded and supernatant used for the TNF*α*-ELISA assays (Abcam), which were performed per the manufacturers instructions. Triplicate samples of 100 *μ*L of each were used for these assays.

### 2.10. C-Reactive Protein ELISA

Serum was obtained from blood harvested via cardiac puncture. The serum samples were centrifuged at 3000 rpm at 4°C for 10 mins using an Eppendorf Centrifuge and stored at −80°C until analysis. Samples were analyzed for the presence of C-reactive protein using an ELISA kit (Abcam) and assays were performed using the manufacturers instructions. Triplicate samples (100 *μ*L) of each sample were used for these assays.

### 2.11. Statistical Analysis

One Way ANOVA and Tukey test, as a post hoc analysis, were used to determine statistical significance between groups using a *P* value of 0.05. Data are expressed as mean ± standard error of the mean (S.E.M) of at least five independent samples as described above. Statistical analysis was performed using GraphPad Prism 6 (GraphPad software).

## 3. Results

### 3.1. Formulating and Characterizing HA-PE Polymers That Form Pericellular Coats

**Figure 1:**
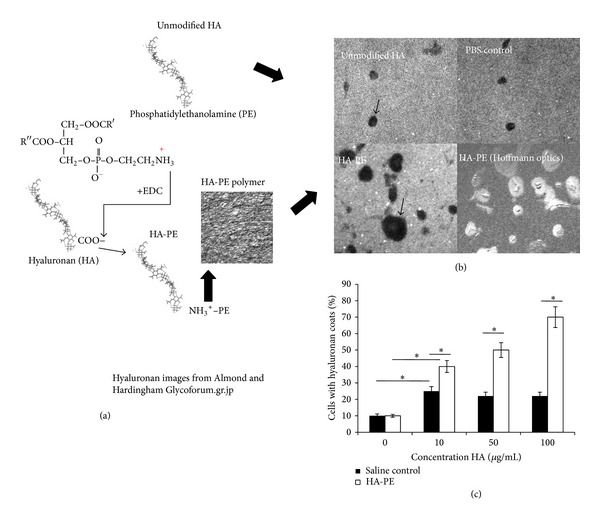
HA-PE simulates pericellular coat formation in cultured fibroblasts. 500 kDa HA was linked to PE using EDC to achieve a stoichiometry of 1 : 10 (HA : PE). (a) This ratio was selected because it resulted in the largest pericellular HA coats, which were detected using fluorescent particle exclusion assays. (b) The black patches on the epifluorescent images are areas of pericellular coats. A Hofmann image is included which shows cells at the center of the pericellular coats. Images were taken with a 10X objective. The percentage cells/10x field that were surrounded by HA coats were calculated using Hofmann optics. (c) Values are Mean and S.E.M *n* = 3 fields. Asterisks indicate statistical significance (*P* < 0.01).

Using a cell-based screen, HA-PE polymer formulations were prepared. The polymer formulations that formed nanoparticles were discarded while those that were nonparticulate and formed pericellular coats on fibroblasts were identified using particle exclusion assays (Figure 1(a)). Addition of HA-PE polymer resulted in a significant increase in the number of cells that formed pericellular coats compared to those formed when PBS alone was added. A light but significant increase in the number of cells forming coats was also stimulated by exogenous unmodified HA of the same size used for HA-PE formulations. Nevertheless, the addition of HA-PE stimulated coat formation to a significantly greater extent than HA alone and a trend to formation of larger coats was also observed (data not shown).

**Figure 2:**
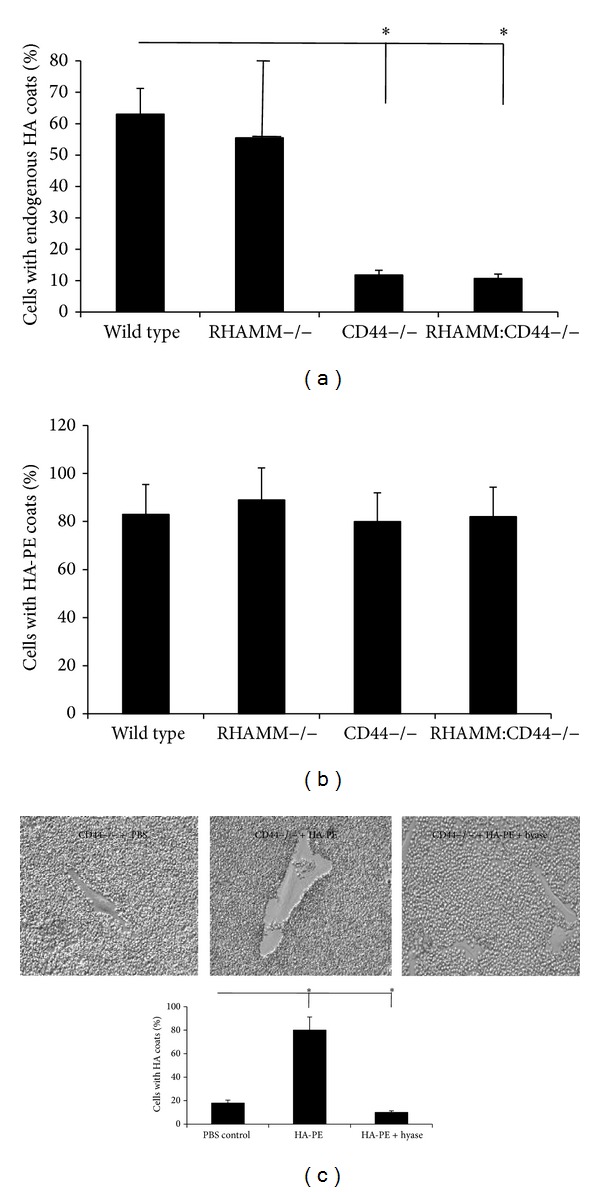
Endogenous HA coats require expression of CD44 while HA-PE generated coats do not. The numbers of wild type and RHAMM−/− fibroblasts that form endogenous HA coats are not significantly different. In contrast, loss of CD44 significantly reduces the numbers of fibroblasts forming coats. This is not further reduced by loss of both CD44 and RHAMM. (a) However, loss of CD44 has no effect on the number of cells forming coats as a result of HA-PE addition. (b) The pericellular coats formed by CD44−/− fibroblasts in the presence of HA-PE are destroyed by hyaluronidase. (c) Images were taken with a 20X Hoffmann objective. Values are the Mean and S.E.M *n* = 75 cells.

CD44 has been demonstrated to be critical for keratinocyte, fibroblast, and smooth muscle cell HA coat formation [32–34, 40, 42] and both CD44 and RHAMM [12, 14, 61] have recently been implicated in the binding of HA to tumor cells and fibroblasts. The role of these HA receptors in the formation of endogenous HA and HA-PE coats was examined by comparing wild type primary mouse embryonic fibroblasts (MEF) with those lacking RHAMM, CD44, or both receptors due to genetic deletion of these genes (Figure 2(a)). Loss of RHAMM resulted in highly variable numbers of MEF forming endogenous pericellular coats but these were not significantly different from wild type fibroblasts. Loss of CD44 however resulted in a 6-fold reduction in the number of MEF exhibiting HA coats. Dual loss of CD44 and RHAMM did not further reduce coat loss from that observed in CD44−/− MEF. These results show that CD44 is the major HA receptor that facilitates endogenous HA coat formation in MEF consistent with previous studies of other cell types including keratinocytes [33, 39, 42]. The effect of HA-PE on RHAMM−/−, CD44−/−, RHAMM : CD44−/−, and wild type MEF coat formation was compared next to begin to identify the mechanisms for HA-PE-promoted pericellular HA coats. As shown in Figure 2(b), the addition of HA-PE to these different fibroblast genotypes resulted in a similar number of cells with HA coats indicating that HA-PE effects were not dependent upon HA receptor display including CD44, which has previously been shown to mediate endogenous coat formation (Figures 2(b) and 2(c)). However, HA-PE stimulated coat formation in CD44−/− MEF was sensitive to hyaluronidase as were endogenous coats (Figure 2(c)). These results predict that addition of a PE group to HA promotes its direct association with cells in a CD44-independent manner.

**Figure 3:**
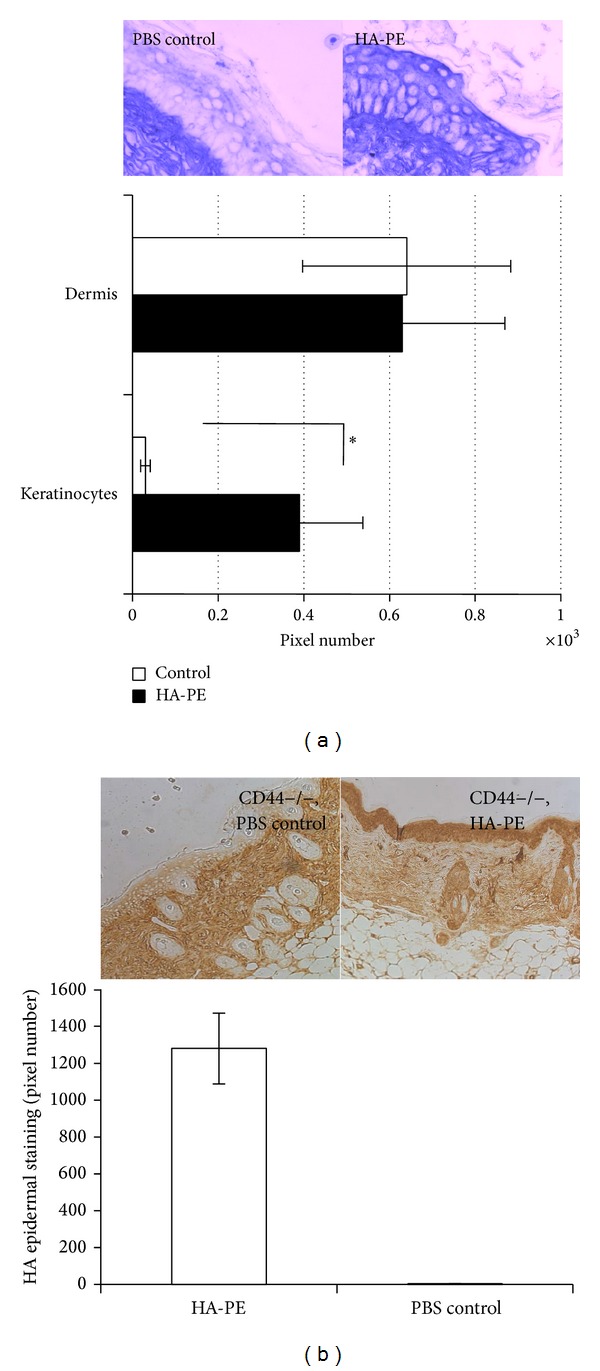
Topical application of HA-PE stimulates HA accumulation in wild type and CD44−/− keratinocytes in mouse skin in vivo. Topical application of HA-PE to shaved B57/BL6 wild type mice increases HA in the keratinocyte layer as detected by biotinylated HABP (blue staining indicates hyaluronan detected by HABP probe). (a) Staining 3 days after application is not detectably different in the dermis and although patchy and variable in the epidermis is significantly different in HA-PE versus vehicle controls (brown staining indicates hyaluronan detected by HABP probe) (*P* < 0.05). Images were taken with a 20X bright field objective. Similar results were obtained when the epidermal layer was analyzed for hyaluronan after HA-PE was applied to B57/BL6 CD44−/− mice. Significantly greater HA staining was observed in HA-PE treated versus vehicle controls (*P* < 0.0001). (b) Images were taken with a 10X brightfield objective. Values in (a) and (b) are the Mean and S.E.M *n* = 3 mice, 5 tissue section/mouse.

To determine if HA-PE promotes coat formation in other cell types particularly in vivo, its effect on skin keratinocyte HA pericellular coats, which have previously been shown to require CD44 expression [30, 42] was next evaluated. Adult mouse back skin was chosen for these studies since it has been reported to produce very little endogenous HA [29], permitting more sensitive detection of exogenous applications of HA-PE using staining methods to detect accumulated HA. Consistent with previous studies [29], very little HA staining was observed in control (PBS) keratinocytes. Topical application of HA-PE to wild type mice significantly increased HA staining in the epidermis (Figure 3) but did not detectably increase HA staining in the dermis when compared to PBS-treated controls likely because the dermis produces large amounts of HA and the staining method was not sensitive enough to detect elevation above this high background. These results show that HA-PE not only enhances pericellular HA coat formation in cultured MEF but also in epidermal keratinocytes in vivo. These results also show that HA-PE crosses the outer cornified epidermal layer more efficiently than unmodified HA.

The association of HA with keratinocytes in mouse skin in vivo depends upon CD44 expression [30]. We therefore next assessed if the loss of CD44 altered HA-PE mediated increases in keratinocyte associated HA in vivo. As shown in Figure 3(b), topical application of HA-PE increased hyaluronan staining of CD44−/− epidermis compared to PBS controls suggesting that epidermal accumulation of HA-PE is CD44 independent in vivo similar to cultured MEF. To more directly follow the association of small amounts of HA-PE with the epidermal and dermal skin layers, we labeled HA-PE with Alexa-dye and analyzed its distribution after a single topical application to mouse skin.

### 3.2. A^647^-HA-PE Accumulates in the Epidermis and Penetrates into the Dermal and Subdermal Skin Layers

**Figure 4:**
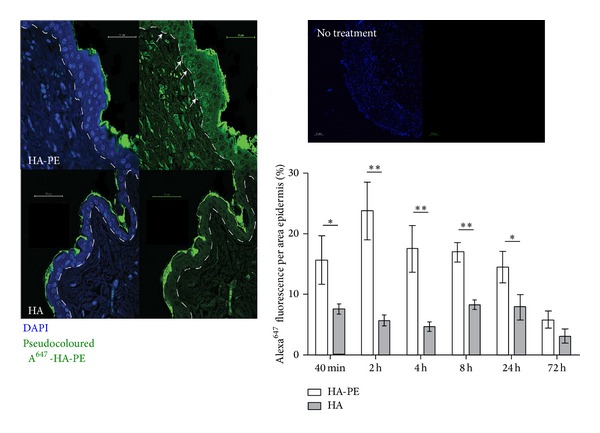
Topical A^647^-HA-PE enters the epidermis. Significantly increased fluorescent staining of A^647^-HA-PE is observed compared to A^647^-HA in the epidermis (stratum basale to stratum granulosum) at all time points up to 24 h (mean ± S.E.M, 20 skin images) (^∗^ *P* < 0.05, ^∗∗^ *P* < 0.001) (right panel). Representative confocal images of A^647^-HA-PE and A^647^-HA treated mice sectioned at 24 h are shown. Merged Dapi and Alexa^647^ staining are shown to indicate cellularity in tissue sections. The dashed line indicates the junction of the epidermis and dermis. Arrowheads indicate areas of enhanced pericellular fluorescence. A^647^-HA-PE and A^647^-HA staining was pseudocolored green. Blue staining is DAPI. An untreated adjacent skin section (no treatment) photographed using the Alexa^647^ channel is shown as a control for autofluorescence.

A^647^-HA-was prepared, linked to PE (A^647^-HA-PE) or not (A^647^-HA) and applied as equal amounts of HA to the shaved back skin of mice as described in methods. Skin biopsy samples were collected from 40 min to 72 h after the single application, processed for histology, and examined with a confocal microscope. Confocal images showed accumulation of A^647^-HA-PE and particularly A^647^-HA on the stratum corneum but A^647^-HA-PE also penetrated into the stratum granulosum as well as the basal keratinocyte layers (Figure 4). Fluorescence was also observed in dermal fibroblasts and even within the subcutaneous muscle layer. Further analysis of the epidermis revealed that A^647^-HA-PE was most strongly associated with subpopulations of basal keratinocytes and formed coats around these cells (Figure 4, arrows). A^647^-HA-PE also was detected in fibroblasts of the upper dermis. In contrast, A^647^-HA primarily accumulated in the outer stratum corneum with much smaller amounts penetrating into the epidermis and dermis. In particular, A^647^-HA accumulation was not concentrated in the basal layer (Figure 4).

Since the boundary of the epidermal layer is clearly identified, the amount of A^647^-HA-PE in this layer was quantified using image analysis as described in methods. For these analyses, the fluorescent intensity of A^647^ HA, A^647^-HA-PE, and a negative control vehicle cream were compared from the stratum basale to stratum granulosum layers of the epidermis. A^647^-HA-PE penetrated into the epidermis as early as 40 min after application and could be detected up to 72 h after application (Figure 4). Accumulation of A^647^-HA-PE in skin was significantly greater than A^647^-HA at all time points but reached a maximum difference of 5 fold at 2 h. This significantly elevated accumulation of A^647^-HA-PE versus A^647^-HA was sustained for 24 h suggesting that HA-PE was able to establish a stable organization within the epidermis. Although A^647^-HA-PE levels were still greater than A^647^-HA at 72 h after application, the difference did not reach statistical significance (Figure 4).

Confocal analysis (24 h shown, Figure 4) showed that A^647^-HA primarily accumulated in the outer stratum corneum with little penetration into the dermis. A^647^-HA-PE was also present in the stratum corneum but unlike unmodified A^647^-HA, accumulated around dermal fibroblasts. These results show that HA-PE readily penetrates into and is retained in the epidermal and deeper skin layers

The formation of pericellular HA coats have been linked to cellular detachment during mitotic rounding of proliferating fibroblasts and smooth muscle cells [12, 16, 81]. HA coats have also been linked to migration and differentiation of keratinocytes in organotypic cultures [4, 13, 14, 30, 82, 83]. We therefore next assessed if increasing keratinocyte HA coat formation by application of HA-PE affects the proliferation or differentiation of this skin cell type.

### 3.3. HA-PE Increases Epidermis Thickness and Basal Keratinocyte Proliferation but Does Not Affect Keratinocyte Differentiation or Dermal Cell Proliferation

**Figure 5:**
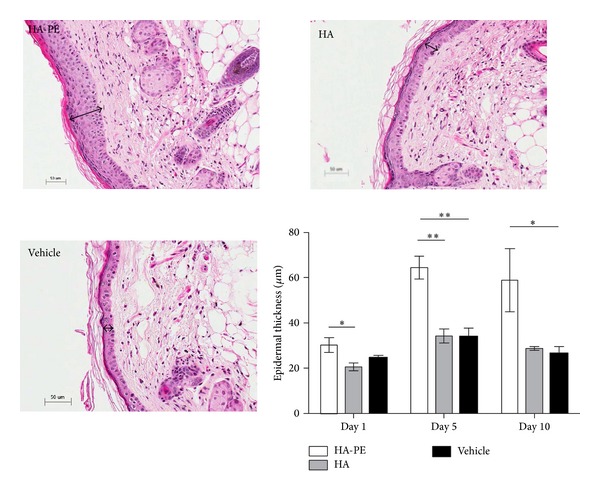
HA-PE increases epidermal thickness. There is a significant increase in epidermal thickness (*μ*m) of mice treated daily with HA-PE for 1, 5, and 10 days compared with control groups (HA and Vehicle cream) (mean ± S.E.M, 5 mice per group) (^∗^ *P* < 0.05, ^∗∗^ *P* < 0.001). A single representative H&E section from each of a day 10 HA-PE, HA, and Vehicle Cream mouse is shown, arrows indicate epidermal thickness.

The consequence of repeated HA-PE topical application on epidermal thickness was first quantified. As shown in Figure 5, daily topical application of HA-PE significantly enhanced epidermal thickness by 24 h, 5 days, and 10 days after treatment initiation when compared to application of unmodified HA or vehicle control. Maximal epidermal thickness occurred between 24 h and 5 days and was sustained throughout the treatment period (Figure 5). The tissue samples were dehydrated prior to analysis of epidermal thickness, and therefore values do not account for any contribution of increased tissue hydration from HA.

**Figure 6:**
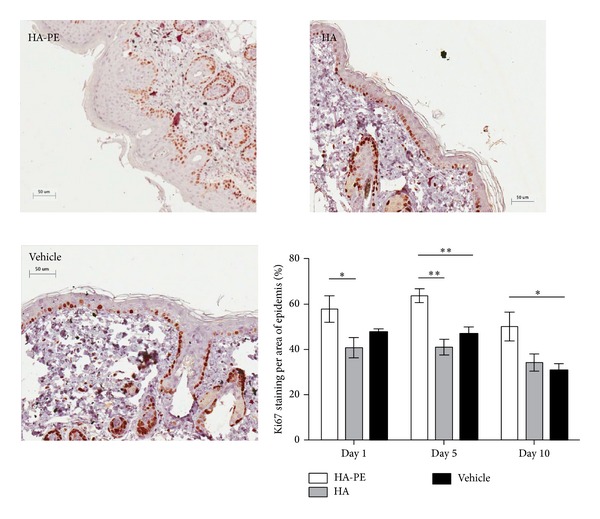
HA-PE stimulates keratinocyte proliferation. There is a significant increase in the percentage of positive Ki67 staining per an area of epidermis of mice treated daily with HA-PE for 1, 5, and 10 days compared with HA or Vehicle Cream (mean ± S.E.M, 5 mice per group) (^∗^ *P* < 0.05, ^∗∗^ *P* < 0.001). A single representative Ki67 section from each of a day 10 HA-PE, HA, and Vehicle Cream mouse is shown.

Consistent with this thickening effect on the epidermal layer, topical application of HA-PE stimulated basal keratinocyte proliferation compared to unmodified HA or vehicle controls as detected by Ki67 staining, which is a cell proliferation marker. In addition, increased Ki67 staining of the suprabasal keratinocytes was observed in the HA-PE group. The majority of epidermal proliferation and regeneration occurs by activation of stem cells in the basal layer of the epidermis. The suprabasal levels contain transit-amplifying (TA) intermediate stem cells which are defined by a finite number of cell divisions before entering a terminal differentiation pathway (85). The HA-PE cream appears to stimulate proliferation of both the basal layer and TA stem cells. Increased keratinocyte proliferation was observed 1 day after treatment initiation and was sustained throughout the treatment period, corresponding well with the time frame observed for the increase in epidermal thickness (Figure 6). Analysis of DAPI- and Ki67 stained skin sections 24 and 72 hr after application of HA-PE versus HA showed that dermal cell number was similar in both treatments indicating that HA-PE does not stimulate fibroblast or other dermal cell proliferation. This predicts that HA-PE effects on proliferation are limited to the epidermal layer.

**Figure 7:**
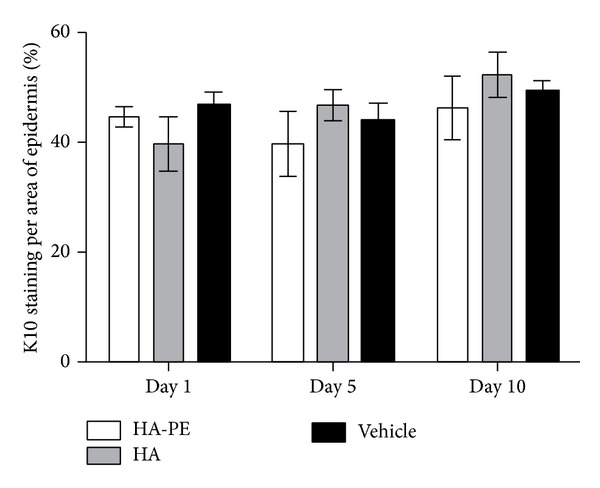
HA-PE cream does not affect suprabasal keratinocyte differentiation. The percentage of K10 staining per an area of epidermis for mice treated daily for 1, 5, and 10 days with either HA-PE, HA, or Vehicle Cream (mean ± S.E.M, 5 mice per group).

Growth factors such as keratinocyte growth factor (KGF) that promote keratinocyte proliferation coincidentally inhibit keratinocyte differentiation and hyaluronan production [84, 85]. We therefore next assessed if elevating HA around keratinocytes affects their differentiation cycle. The consequence of HA-PE application on the expression of keratin-10 (K-10) was quantified using immunohistochemistry. No detectable effect of HA-PE application on K-10 staining was observed suggesting that HA-PE does not modify keratinocyte differentiation (Figure 7).

### 3.4. Topical Application of HA-PE Does Not Affect Local Skin or Systemic Inflammation

**Figure 8:**
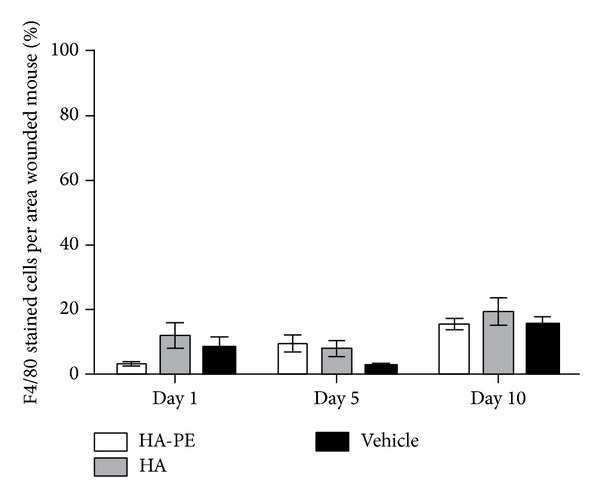
HA-PE cream does not elicit a local inflammatory response. The percentage of positive F4/80 staining per an area of a wounded mouse for mice treated daily for 1, 5, and 10 days with either HA-PE, HA, or Vehicle Cream (mean ± S.E.M, 5 mice per group).

**Figure 9:**
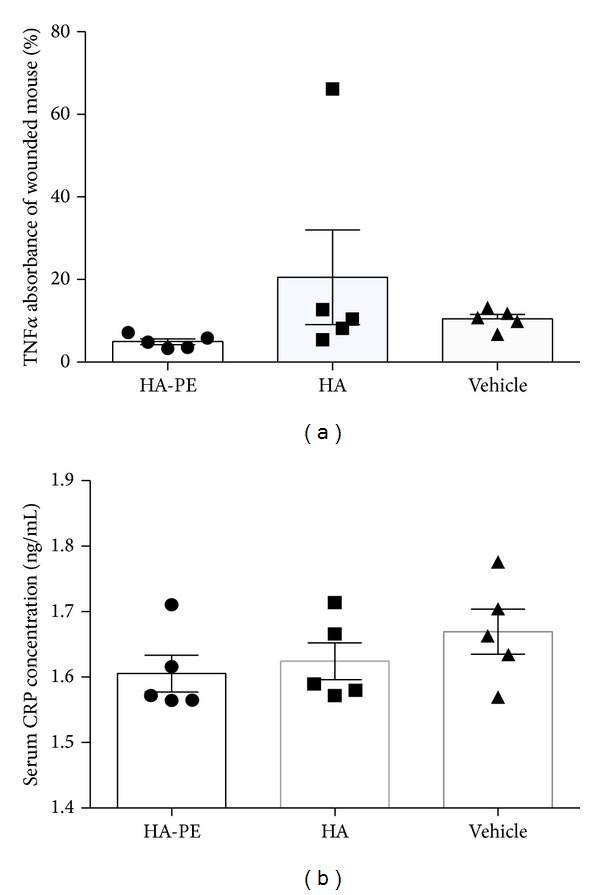
HA-PE cream does not elicit a local or systemic inflammatory response. The TNF*α* expression as a percentage of a wounded mouse control for day 10 treated mice with HA-PE, HA, or Vehicle Cream using an ELISA (mean ± S.E.M, 5 mice per group). (a) The serum CRP concentration (ng/mL) of day 10 treated mice with HA-PE, HA, and Vehicle Cream using an ELISA (mean ± S.E.M, 5 mice per group) (b).

Since HA and its fragments are potent regulators of the immune system in particular innate immunity [15, 19, 46, 86], which could indirectly affect keratinocyte proliferation, we next determined if topical application of HA-PE affected local or systemic inflammation. F4/80 staining of skin tissue sections and TNF*α* was used to identify localized activation of macrophages while serum CRP levels were monitored to determine effects on systemic inflammation. As shown in Figure 9, the application of HA-PE did not increase F4/80 staining levels compared to unmodified HA or vehicle controls (Figure 8). Furthermore, the levels of macrophage activation detected by this method were very low compared to those detected in skin wounds, which are known to contain high levels of activated macrophages [87] and were used as a positive control. Similarly local TNF*α* and systemic CRP inflammation levels were not increased by topical application of HA-PE compared to unmodified HA or vehicle controls (Figures 9(a) and 9(b)).

## 4. Discussion

Our results identify a method for preparing HA-phospholipid (PE) polymers that do not require CD44 display for forming pericellular coats around fibroblasts in culture and aged keratinocytes in wild type and CD44−/− mice. These results are consistent with a mechanism whereby HA-PE inserts directly into the cell membrane via the phospholipid entity. The topical applications of this HA-PE polymer increased epidermal thickness of aged female mouse skin as a result of stimulating the proliferation of the basal keratinocyte layer in the absence of detectable dermal or systemic inflammation or changes in keratinocyte differentiation rates.

Topical application of unmodified HA to intact skin in situ penetrated into the epidermis and dermis as previously reported [42, 69–71] but this was limited compared to HA-PE polymers. In addition, most of the unmodified HA remained associated with the stratum corneum and was not retained in skin as long as HA-PE. The ability of unmodified HA to penetrate the skin in small amounts, which can occur in both rodent and human skin, is dependent upon molecular weight. Thus, HA polymers smaller than 50,000 daltons readily penetrate human skin ex vivo while larger HA chains do not [69]. The average MW of HA used in the present study was 500,000 Da and therefore the limited association of unmodified HA with the epidermis is likely due to the polymer size restriction. The penetration of this HA size through the outer stratum corneum is facilitated by the addition of a phospholipid moiety and this addition likely increases solubility of HA in lipids (e.g., ceramides, cholesterol, and fatty acids) [88] that are present in the stratum corneum. Retention within the epidermis and dermis is likely promoted by the ability of keratinocytes and fibroblasts to capture and retain HA-PE as demonstrated in culture. Since HA-PE is able to organize as a pericellular coat in the absence of CD44 display, HA-PE polymers are able to spontaneously organize within the epidermis and we predict that keratinocyte CD44 acts at least in part as an anchor for attaching HA to outside of cells.

Dermal and epidermal homeostasis is compromised in aging and a variety of disease processes. Aged skin is characterized as dry, atrophic, inelastic, and wrinkled. The key molecule required for water retention within skin is HA. Both the amounts and organization of HA change in skin with intrinsic (chronological) and extrinsic (photodamage) aging [4, 89, 90]. With chronological aging while HA is retained within the aged dermis, it is precipitously lost from the epidermis. HA retained within the aged dermis is however modified in its organization and this change together with epidermal loss are thought to contribute to some of the above malfunctions of aged skin [4]. Additionally, expression of skin HA receptors including CD44 are increasingly reduced with age. Even though intrinsic and extrinsic skin aging are distinctive processes, they share similarities in molecular mechanisms. For example, extrinsic causes of premature aging such as chronic exposure to UV also results in loss of skin moisture, HA and HA receptor expression. Importantly, photoaging is also associated with a decrease in the size of HA that will likely affect its ability to organize into structures such as pericellular coats [72]. HA performs a variety of functions in skin and this age dependent loss is considered to impact upon skin moisture, barrier functions, epidermal thinning, and sluggish response to injury [34]. The ability of HA-PE polymers to increase rodent epidermal thickness and epidermal proliferation predict that this formulation has the potential for reversing some of the intrinsic and extrinsic age-related epidermal defects such as epidermal thinning and reduced repair response [91–93].

Our results showing that HA-PE promotes epidermal thickening and keratinocyte proliferation are similar to the ability of exogenous high molecular weight HA to affect functions of aged keratinocytes in culture and in vivo [33, 35, 39, 42] although in the present study topical HA-PE had a significantly greater effect than unmodified HA. Our data suggest that this results from increased basal keratinocyte proliferation rather than enhanced differentiation. Previous reports have similarly noted an increase in keratinocyte proliferation in response to topical HA but noted this effect was maximal for intermediate (50,000–400,000 Daltons) and no effect was noted for large HA (>400,000 Daltons) [39]. In another study, topical application of large HA promoted epidermal barrier function and keratinocyte differentiation [42]. The different effects of high versus intermediate HA fragments in these studies may have been due to the documented poor penetration of high molecular weight HA [69] that is confirmed here. The ability of 500,000 dalton HA-PE to promote keratinocyte proliferation is likely because it efficiently penetrated to and was retained in the basal keratinocyte layer to a much greater extent than unmodified HA.

A number of topical HA formulations have been developed in the past decade most of which are composed of intermediate-small HA fragments (e.g., <50,000 daltons) or nanoparticulate formulations [4, 94]. Although these have shown efficacy (e.g., [95]) in terms of treating skin disorders, we pose that HA-PE formulations will be more effective since they are designed to replace the naturally occurring HA pericellular coat that is depleted during aging and other conditions that cause epidermal atrophy [35, 39] and will also therefore be retained within the epidermis for longer times. Particulate formulations by their nature tend to stimulate endocytosis and such formulations are rapidly depleted from tissues [96–98]. Topical application of intermediate unmodified HA has the advantage of being nonparticulate and able to penetrate skin readily but is dependent upon the expression of keratinocyte CD44 for effects on keratinocyte function, which is depleted with age-related or other causes of epidermal atrophy.

The consequences of exogenous HA or inhibition of endogenous HA show that CD44 mediates the consequences of HA on keratinocyte function [4, 6, 60, 99]. For example, the effects of topical HA on keratinocyte differentiation and proliferation are ablated when CD44 function is blocked or expression is lost [35, 39, 42]. CD44 appears to regulate multiple downstream signaling pathways to control these keratinocyte processes and these include coordination of signaling through EGFR, activation of RHO GTPase and translocation of activated ERK1,2 to the cell nucleus [4, 17, 35, 99, 100]. The mechanisms by which HA-PE control basal keratinocyte proliferation and the dependence of this effect on CD44 expression are currently being investigated.
